# Inaudible and invisible: epistemic injustice and the failure of recognition as a single intersubjective relation. Pain as particularly illuminating case

**DOI:** 10.1007/s11019-026-10371-1

**Published:** 2026-07-14

**Authors:** Helmar Bornemann-Cimenti

**Affiliations:** Department of Anaesthesiology and Intensive Care Medicine, Medical University Graz, Graz, Austria

**Keywords:** Epistemic injustice, Recognition, Chronic pain, Fricker, Honneth, Clinical encounter

## Abstract

Miranda Fricker’s concept of epistemic injustice and Axel Honneth’s theory of recognition have each been applied, separately, to chronic pain. The relationship between them is usually construed as sequential: epistemic injustice is thought to produce failures of recognition, or prior misrecognition to ground later epistemic marginalisation. This article rejects that reading. It argues that, in the clinical encounter with a person in pain, the relation between the two is not exhausted by causal succession. They are analytically separable dimensions of a single intersubjective relation, dimensions that cannot, in that encounter, be treated apart without descriptive and practical loss. The act by which a clinician withholds credibility from a patient’s testimony is the same act by which recognition is refused to that patient as a suffering subject. The claim is offered as a heuristic rather than an ontological thesis. Pain is presented not as uniquely paradigmatic but as a particularly illuminating case within a broader class of testimonially dependent suffering, since its privacy, its dependence on testimony, and the mediating role of diagnostic language converge in it with unusual clarity. The argument bears on recognition theory; on medical ethics, where remedies addressed to one dimension alone prove insufficient; and on clinical practice, where diagnostic classification operates as a technology of recognition.

## Introduction: pain as a philosophical laboratory for intersubjectivity

Few human experiences expose the fragility of intersubjective relations as starkly as pain. The sufferer is caught in a peculiar double bind: the experience is overwhelmingly certain to the one who has it, yet it remains, in Scarry’s formulation, resistant to the language through which it might be shared.(Scarry 1987) This asymmetry between first-personal certainty and third-personal opacity places pain at the intersection of epistemology and ethics. To be in pain is to depend, existentially, on the willingness of others both to believe one’s report and to acknowledge one’s suffering as worthy of response. When either is withheld, the consequences extend beyond inadequate analgesia; they bear on the conditions under which a person can sustain a coherent practical identity.

Two philosophical traditions furnish vocabularies for analysing these failures. Fricker’s account of epistemic injustice identifies the wrong done to a person specifically as a knower, whether through prejudicial credibility deficits (testimonial injustice) or through gaps in the collective resources available for interpreting one’s experience (hermeneutical injustice). (Fricker 2007) Honneth’s theory of recognition, rooted in a Hegelian tradition, identifies three spheres, love, rights, and solidarity, in which acknowledgement is required for the self-relations of self-confidence, self-respect, and self-esteem.(Honneth 1996) Both have been brought to bear on chronic pain: Buchman, Ho and Goldberg applied Fricker’s framework to pain management, while Koesling and Bozzaro adapted Honneth’s recognition theory to the social struggles of pain patients.(Buchman et al. 2017; Koesling and Bozzaro 2021) These bodies of scholarship have developed largely in parallel, and where they have been connected the link has been read as sequential: epistemic injustice is taken to produce failures of recognition, or recognitive failure to generate the conditions for epistemic marginalisation. In a 2018 special issue of *Feminist Philosophy Quarterly*, Fricker herself gestured towards extending Honneth’s modes of recognition to yield an epistemically specific form.(Fricker 2018). That suggestion has not been developed into a systematic account of the structural relationship between the two phenomena, nor tested against a concrete domain of experience.

The present article takes up that task. Its central claim is that, in the clinical encounter with a person in pain, epistemic injustice and the failure of recognition are not merely sequential phenomena but analytically separable dimensions of a single intersubjective relation that cannot be addressed apart without descriptive and practical loss. In the single testimonial act, the withholding of credibility from a sufferer’s testimony is the mode in which the failure of recognition is enacted, just as the denial of recognition is, in that same act, already an epistemic deprivation. That one phenomenon may, across encounters and over time, causally facilitate the other is not denied, and is taken up in Sect. ''The Sequential Model and Its Limitations''. The claim is advanced as a heuristic rather than a metaphysical thesis, leaving open whether the two dimensions are, at some deeper level, one. Pain is treated not as uniquely paradigmatic but as a particularly illuminating case within a broader class of testimonially dependent suffering, the case in which the structure is least obscured by confounding factors. Three stakes follow, developed below: that, for this class of encounters, epistemic injustice and recognition cannot be assigned to separate philosophical registers; that clinical remedies addressed to only one of the two dimensions will, for that reason, fall short; and that the institutional binding of recognition to diagnostic classification, and not the individual act of belief alone, is a principal locus at which reform must be directed.

## Epistemic injustice: Fricker’s framework and its application to pain

Fricker’s account locates a distinctive species of wrong in the treatment of persons specifically in their capacity as knowers. She distinguishes two forms. Testimonial injustice occurs when a hearer, under the influence of a prejudice attaching to the speaker’s social identity, assigns the speaker’s word a credibility lower than it merits. The prejudice, rather than any defect in the testimony itself, does the deflating work; identity prejudice is therefore a necessary condition of the wrong and not a mere aggravating circumstance.(Fricker 2007) Hermeneutical injustice, by contrast, is structural rather than transactional: it arises when a lacuna in the collective interpretive resources of a community leaves a region of someone’s experience unintelligible, so that the subject cannot render their own situation communicable even to themselves. The two forms are independent in principle, yet they frequently compound one another, and the clinical setting supplies conditions under which both flourish. A patient’s report of pain is at once a testimonial claim, exposed to prejudicial discounting, and an attempt to deploy interpretive categories that may be unavailable or ill-fitted to the experience at hand. The application of Fricker’s apparatus to medicine therefore confronts, from the outset, a domain in which the testimonial and the hermeneutical are difficult to prise apart.

Feminist epistemology has extended this framework in directions that bear directly on the pain encounter. Dotson identifies a form of self-imposed testimonial restriction she calls testimonial smothering, in which a speaker, anticipating that their testimony will be misconstrued or rejected, truncates or withholds it in advance; the silence is coerced by the hearer’s demonstrated incompetence or hostility rather than freely chosen. (Dotson 2011) She later analyses a deeper, third order of epistemic oppression, one that is irreducible to credibility deficits or hermeneutical gaps because it consists in the resistance of a dominant epistemic system to the alternative resources that marginalised knowers have in fact produced.(Dotson 2014) Both phenomena recur in chronic pain care. Patients who have learnt, across repeated consultations, that articulating the full extent of their suffering invites scepticism come to ration their disclosures. The experiential vocabularies they generate among themselves seldom penetrate the categories through which clinicians are trained to listen. Dotson’s analysis thus shows that the epistemic marginalisation of the person in pain is not exhausted by the moment at which a single report is disbelieved; it encompasses the anticipatory silences and the unabsorbed counter-vocabularies that surround that moment.

The transposition of these ideas to medicine was accomplished most influentially by Carel and Kidd, whose work in this journal established illness as a paradigmatic site of epistemic injustice. (Carel and Kidd 2014) They argued that ill persons are subject both to characteristic credibility deficits, their first-personal reports routinely subordinated to third-personal and instrumental evidence, and to hermeneutical marginalisation, the categories of biomedicine being poorly suited to the existential texture of illness. The argument was deepened in subsequent work specifying the mechanisms by which the testimonies of ill persons are pre-emptively downgraded.(Kidd and Carel 2017) It rests on a phenomenological account of illness as a transformation of the lived body and its world, one that resists assimilation to the naturalistic categories of the clinic. (Carel 2016) The same framework has been carried into psychiatry, where the attribution of disordered cognition supplies an especially potent ground for testimonial discounting.(Crichton et al. 2017) This extension is instructive for the present analysis, since it marks the outer boundary of the class of cases to which the argument offered here belongs, a boundary revisited in Sect. ''Pain as a Particularly Illuminating Case''.

Within the philosophy of pain specifically, Fricker’s categories have been applied with increasing precision. Buchman, Ho and Goldberg showed that the narratives of chronic pain sufferers are systematically downgraded in clinical encounters, the credibility deficit tracking patients across consultations and institutions and intensifying where social identities intersect. (Buchman et al. 2017) Tosas located the source of this downgrading in the epistemological commitments of biomedicine itself, which privilege objectively verifiable pathology over subjective report.(Tosas 2021) The pattern has been documented across particular conditions: in the protracted effort of women with endometriosis to have their pain communicated and believed (Bullo 2020), and in the epistemic dimensions of menstrual pain, examined in this journal. (Mickiewicz 2025) Empirical work has begun to specify the social contours of the deficit, demonstrating identity-prejudiced disparities in the assessment and treatment of pain along lines of race (Hoffman et al. 2016) and of sex (Guzikevits et al. 2024); these findings are taken up in detail in Sect. ''The Co-Constitution Thesis''. A recent qualitative study of patients with what the literature terms ‘medically unexplained’ pain traced the trajectory by which repeated dismissal hardens into anticipatory silence and eventual withdrawal from care, with testimonial and hermeneutical injustice shown to be mutually reinforcing rather than merely co-present.(Okuhara et al. 2026).

The application of Fricker’s framework to pain has not gone uncontested, and two recent interventions sharpen the conceptual terrain in ways the present analysis must accommodate. De Souza Campos and De Luca-Noronha argue that a distinct species of epistemic failure operates in chronic pain reporting, which they term distributive epistemic injustice: a failure to distribute inquisitive attention fairly, manifested as inquisitive inertia, the clinician’s disinclination to ask the further questions that would elicit a fuller account. They are explicit that this wrong can obtain even where neither testimonial nor hermeneutical injustice is present, so that it is not reducible to the two Frickerian forms.(De Souza Campos and De Luca-Noronha 2025) Wiggleton-Little presses a complementary worry from the opposite direction, contending that some dismissals of pain reflect a pain-related motivational deficit that the vocabulary of credibility cannot capture, so that the epistemic-injustice framework overreaches when made to absorb every failure of uptake. (Wiggleton-Little 2025) Both objections are accommodated here, for the heuristic reading developed below does not claim that the epistemic categories exhaust the wrongs at stake, only that, in the clinical encounter, the epistemic and the recognitive dimensions cannot be treated separately without descriptive loss. A more searching reservation has been voiced by Nielsen, Nordgaard and Henriksen, who question the conceptual coherence and empirical grounding of the epistemic-injustice-in-medicine literature as a whole. (Nielsen et al. 2025) This critique has itself been contested: in a reply published in this journal, Færge and colleagues argue that to require quantitative operationalisation before the concept is granted clinical and ethical significance risks reinstating the very hierarchy of knowledge that epistemic injustice was meant to expose, and they urge in its place a methodological pluralism suited to the phenomenon. (Færge et al. 2026) The exchange is registered here without adjudication; the field is conceptually heterogeneous and its empirical base uneven, yet it remains in active philosophical and empirical development. The present analysis is offered not as a defence of the field in its current form but as a contribution to its sharpening, by identifying and correcting at least one persistent source of imprecision: the unexamined assumption that the epistemic and the recognitive are separable phenomena.

## Recognition and its failure: Honneth’s three spheres in the pain context

Honneth’s theory of recognition, developed in *The Struggle for Recognition* and refined in subsequent work, identifies three modes of intersubjective acknowledgement constitutive of the self-relations a flourishing life requires. In the sphere of love and primary relations, recognition takes the form of emotional concern, enabling basic self-confidence. In the sphere of rights, it consists in the attribution of moral responsibility and legal standing, sustaining self-respect. In the sphere of solidarity, it involves the social esteem accorded to an individual’s particular capacities and contributions, grounding self-esteem. Each sphere has its corresponding form of disrespect: the violation of bodily integrity, the denial of rights, and the denigration of ways of life.(Honneth 1996).

Koesling and Bozzaro have undertaken the most systematic adaptation of this framework to chronic pain to date.(Koesling and Bozzaro 2021) They show that pain patients are vulnerable to misrecognition in all three spheres, though the forms of disrespect require modification. In the sphere of primary relations, the disrespect seldom takes the form of physical violence, the paradigmatic case in Honneth’s original account; more often it is the withdrawal of empathic attentiveness by intimate others who grow weary of a suffering that resists resolution. In the sphere of rights, patients confront the erosion of their standing as autonomous agents when their self-reports are overridden by clinical authority or when bureaucratic gatekeeping denies them access to treatment. In the sphere of solidarity, the invisibility of chronic pain within the social imaginary undermines the esteem that would ordinarily attach to the endurance of adversity.

Honneth has himself recently addressed the relationship between his framework and Fricker’s. (Honneth 2022) He observes that testimonial injustice corresponds most closely to disrespect in the sphere of rights, the denial of a person’s standing as a morally responsible agent, whereas hermeneutical injustice, lacking an identifiable perpetrator, fits the recognitional scheme less readily. Honneth’s alignment locates testimonial injustice in a single sphere. The recognitive failures that attend the disbelief of a person in pain, however, range across all three: they erode the basic self-confidence of primary relations, contest the standing of the patient as a responsible agent, and withhold the esteem that solidarity would confer. The *recognitive* dimension at issue in the present analysis is therefore not to be identified with any one Honnethian sphere, but cuts across them wherever the reception of testimony is at stake. More consequentially, he argues that the two are not merely adjacent but internally linked: to deny a person the standing of a responsible agent is at the same time to deny them the capacity to make truth-apt claims, so that, in his words, “moral disrespect … immediately implies epistemic disrespect”.(Honneth 2022) From this he draws a directed conclusion. What he terms *epistemic recognition*, the disposition Fricker analyses as testimonial sensibility, is a secondary component of what he terms *moral recognition*. Moral recognition is the more fundamental, since it settles in advance whose cognitive credibility is to be weighed within a relationship at all. These terms are Honneth’s own and are used here strictly in his sense; they are to be kept distinct from the *epistemic* and *recognitive* dimensions that the present analysis ascribes to the clinical encounter.

Two consequences follow for the present argument. First, Honneth’s treatment, like Koesling and Bozzaro’s, proceeds at the level of conceptual and normative theory; neither examines how the relation between the epistemic and the recognitive is structured within the concrete clinical encounter, where a patient’s testimony is received, weighed, and answered in real time. Second, and more delicately, Honneth’s asymmetry might seem to count against a thesis that speaks of co-constitutive dimensions of a single relation, a formulation open to being read as a claim to parity. It does not. Honneth’s priority runs in the order of grounding, from the moral to the epistemic; the order of enactment in the testimonial encounter runs the other way, since moral recognition cannot be extended to a person in pain except through the uptake of the very report whose credibility is in question. A hierarchy among concepts thus coexists with an inseparability among their enactments, the two claims being pitched at different levels (developed in Sects. ''The Sequential Model and Its Limitations'' and ''For Social Philosophy''). In establishing that moral disrespect implies epistemic disrespect, Honneth establishes one half of what is needed to show that the two cannot vary independently, and it is to that question of independence that the analysis now turns.

## The sequential model and its limitations

The relationship between epistemic injustice and the failure of recognition is standardly construed as sequential, whether explicitly or as an unexamined background assumption, though the direction of the sequence is disputed. On one account, epistemic injustice is prior: a patient’s testimony is dismissed, or the patient lacks the resources to articulate their experience, and this epistemic marginalisation then produces a failure of recognition, the patient ceasing to be acknowledged as a competent participant in the practices through which esteem and respect are distributed. On the alternative account, the failure of recognition is prior: because the patient is not recognised as a full member of the relevant community, whether on account of race, gender, socioeconomic status, or the stigma attaching to chronic pain, their testimony is subsequently devalued as a downstream consequence of social exclusion.

Each version captures something real. There are indeed cases in which one phenomenon temporally precedes or causally facilitates the other. A patient first denied recognition as a morally autonomous agent, through the paternalistic overriding of their treatment preferences, may afterwards find their pain reports taken less seriously, the loss of moral standing contaminating epistemic credibility. Conversely, a patient whose testimony is persistently discounted may over time internalise a diminished sense of self-worth amounting to a failure of the self-relation Honneth locates in the sphere of solidarity. (Honneth 1996) To this extent the sequential model is not mistaken, and an adequate account must preserve its insights rather than discard them.

The model becomes vulnerable only when the sequence is taken to license a stronger inference about the independence of the two phenomena. It might be supposed that, if the epistemic and the recognitive are distinct domains joined by contingent causal pathways, then it should be possible in principle to encounter epistemic injustice in the absence of any recognitive failure, and conversely. The inference moves too quickly. A causal or sequential model may hold that one phenomenon activates, deepens, or structures the other without thereby committing itself to the claim that either can occur alone. The label ‘sequential model’ in fact conflates four distinct theses: (a) that one phenomenon temporally succeeds the other; (b) that one causally explains the other; (c) that the two are ontologically separable, belonging to independent domains; and (d) that each can occur in the absence of the other. These theses are logically independent, and a proponent may affirm some while denying others.

With the theses separated, the target of the present critique can be specified, and it is narrower than the rhetoric of the sequential model suggests. A word on terminology is needed first, since the name of the position invites misreading. The label *co-constitution* names the thesis as a whole. It stands for the heuristic claim defended below: that the epistemic and the recognitive co-vary and, issuing from a single source, cannot be treated apart without descriptive and practical loss. It does not assert that the two are identical, mutually reducible, or metaphysically constitutive of one another. Within that thesis, *co-variation* names the narrower logical result that the two do not occur independently, and *inseparability* the operative claim that they cannot be described or remedied apart. With the term so fixed, the co-constitution thesis does not contest (a) or (b); temporal succession and causal facilitation are real and may be granted without reservation. Nor does it undertake to refute (c) on its own, ontological terms. Its two claims are these: that (d) is false, the two not in fact occurring independently across the class of encounters at issue; and that (c), whatever its ultimate truth, is the wrong frame in which to describe those encounters.

Claim (d), that the two can occur independently, is the more readily answered, and answering it requires both directions of implication. Honneth supplies one direction, though its reach must be stated with care. His claim bears on disrespect in the sphere of rights: to deny a person the standing of a morally responsible agent immediately implies a denial of their capacity to make truth-apt claims.(Honneth 2022) In the testimonial encounter this is the operative component, since to disbelieve a person’s report of their own pain contests their standing as a reliable witness to their own condition; for recognitive failures of this kind, no failure of recognition occurs, in this class of cases, without a corresponding epistemic wrong. The implication is not asserted for failures bearing solely on the spheres of love or of solidarity, and need not be, since the converse direction does not rest on it. That converse direction, that no withholding of credibility occurs in this class without a corresponding refusal of recognition, is established not by Honneth but by the structure of the testimonial act examined in Sect. ''Testimony and the Indivisibility of Credibility and Care''. There the assessment of a pain report and the recognition of its author prove not to be separately performable. Taken together, the two directions yield co-variation across the class of testimonially dependent encounters at issue. This is co-variation, not identity: to show that the two cannot occur independently is not to show that they are the same thing, but that the sequential model’s strongest inference, from the distinctness of two domains to their possible independence, does not go through.

Claim (c) is the assumption that the epistemic and the recognitive are independent domains, contingently linked. This the analysis does not attempt to defeat at the level of metaphysics; it brackets the question whether the two are, at some deeper level, one. The objection to (c) is heuristic rather than ontological, and co-variation alone would not sustain it, for two phenomena that always occur together might still be described, and remedied, separately. What carries the heuristic claim is a further and more contestable premise, defended in Sect. ''The Co-Constitution Thesis'', on which the burden of the argument finally rests: that in the clinical encounter the two issue from a single judgement of the patient and a single act of reception. This is not a restatement of the inseparability it is meant to ground but an independent claim about the phenomenology of the testimonial act, namely that there is in that act no moment at which credibility is weighed apart from the question of recognition. If the premise holds, then the two dimensions share this root. Treating them as separable domains therefore exacts a cost in descriptive adequacy and in clinical efficacy, and no remedy directed at the one in abstraction from the other can reach the judgement from which both proceed. The constructive thesis is accordingly conceptual and heuristic: resting on the co-variation just established and on this single-source structure, it asserts that the analytic separation of the two dimensions misdescribes the encounter and misdirects its remedies, without requiring that the dimensions collapse into one. These grounds are set out in the next section; Sect. ''Pain as a Particularly Illuminating Case'' then explains why pain discloses the structure with particular clarity.

## The co-constitution thesis

The central claim of the present analysis is that, in the context of pain, epistemic injustice and the failure of recognition are analytically separable dimensions of a single intersubjective relation which cannot be treated apart, in the clinical encounter, without descriptive and practical loss. The relation in question is the encounter between a suffering subject and another who is positioned to receive, interpret, and respond to the testimony of suffering. The thesis is defended on three grounds, corresponding to three dimensions of that encounter: the act of testimony, the mediating role of diagnostic language, and the structure of the clinical relationship.

### Testimony and the indivisibility of credibility and care

When a person in pain offers testimony, in the bare report ‘I am in pain’, they perform an act that is at once epistemic and recognitive. Epistemically, they advance a claim about their own experiential state, a claim that invites assessment of its credibility. Recognitively, they make a bid for acknowledgement, presenting themselves as a subject whose suffering matters, whose first-personal authority over their experience deserves respect, and whose vulnerability calls for a caring response. In this act the two are not separately performable. To assess the credibility of a pain report is already to take a stance on the kind of subject the speaker is, and to extend or withhold recognition from a suffering person is already to have made a determination about the reliability of their self-report. The point is not metaphysical but descriptive: in the testimonial encounter there is no neutral moment at which credibility is weighed before, or apart from, the question of recognition.

The phenomenology of testimonial failure makes this plain. In the study of patients with medically unexplained pain, repeated dismissal did not merely lower the patients’ epistemic standing; it eroded the basic self-confidence that, on Honneth’s account, depends on recognition in the sphere of primary relations.(Okuhara et al. 2026) Patients reported not simply being disbelieved but being rendered invisible, a description that leaves no room for the distinction between epistemic marginalisation and recognitive denial. The experience of not being heard is not an epistemic event succeeded by a recognitive consequence; it is given as one, the loss of credibility and the loss of standing arriving together and indistinguishable as lived.

Evidence from research on racial bias in pain assessment supports the same reading. Hoffman and colleagues found that a substantial proportion of White medical students and residents in the United States held false beliefs about biological differences between individuals racialised as Black and White, for instance that Black skin is thicker or that Black people feel less pain. These beliefs predicted both lower pain ratings and less accurate treatment recommendations for Black patients. (Hoffman et al. 2016) The false beliefs are not epistemic errors that merely happen to issue in recognitive consequences. They are, at one and the same time, identity-prejudiced credibility deficits of the kind Fricker describes and denials of the equal standing of Black patients as subjects whose suffering warrants the concern extended to White patients. That a single false belief should issue in both at once might be read as mere common causation, one cause with two effects that remain in themselves separable. That picture of two downstream effects should be resisted. In applying the belief, the clinician forms a single judgement of the kind of patient before them; the epistemic deflation and the recognitive denial are aspects of that one act rather than two products of it. No intervention can therefore correct that act under the one description while leaving it, under the other, in place.

### Diagnostic language as epistemic and recognitive medium

The clinical encounter is not conducted in natural language alone. It is mediated by a dense apparatus of diagnostic categories, classification systems, and measurement instruments, from the International Classification of Diseases to the Numerical Rating Scale, from the McGill Pain Questionnaire to the criteria for conditions such as fibromyalgia or complex regional pain syndrome. The assignment or withholding of a diagnosis is a moment within the clinical encounter rather than an encounter apart from it, but it is the moment at which the epistemic and the recognitive converge most sharply, since it is here that the patient’s testimony is translated into an institutional category, or fails to be. The role of this apparatus is easily misstated, and three claims must be held apart: a descriptive claim about how diagnosis functions in contemporary practice, a mechanistic claim about why pain is especially exposed to its workings, and a normative claim about how recognition ought, or ought not, to be conditioned upon it.

Descriptively, the diagnostic label functions, in contemporary medicine, as a technology of recognition. To receive a recognised diagnosis is to acquire the social standing of someone who is genuinely ill, whose suffering is legible, and who has a claim upon care. It is also to be admitted to what Parsons called the sick role (Parsons 1951): the sanctioned exemption from ordinary obligations, the licence to rest and to withdraw from work and social duties without suspicion, and, in many systems, the access to material support that legitimate illness confers. A patient whose pain maps onto no recognised category, the patient whose pain corresponds to no identifiable lesion or biomarker, is by contrast doubly dispossessed. The same institutional fact appears, under one description, as a hermeneutical lacuna of the kind Fricker analyses and, under another, as the denial of esteem that Honneth locates in the sphere of solidarity.(Honneth 1996; Fricker 2007) Diagnostic categories, moreover, do not merely register the experiences they name; they reshape them. The looping effect by which classification alters the conduct and self-understanding of those it classifies (Hacking 1996) operates at the nosological level in pain medicine as elsewhere, where the availability of a label can consolidate, and its absence can corrode, the very experience it purports to describe. (Gauld et al. 2025) All of this describes how the system works, not how it ought to.

Why pain should be so exposed to this mechanism becomes clear once the epistemological commitments of biomedicine are made explicit. The dominant model treats disease as the manifestation of an underlying lesion, a localisable tissue pathology or, in more recent idiom, a measurable biomarker. The lineage of this lesion-based conception runs through the anatomical pathology of Bichat and his nineteenth-century successors, and despite the long-standing case for a biopsychosocial alternative (Engel 1977), it remains the tacit standard against which claims to illness are measured. Pain that answers to this standard, the nociceptive pain accompanying identifiable tissue damage, secures a surrogate objectification, for the lesion vouches for the report. Pain that does not is left without such warrant. Nociplastic pain is the category introduced to name pain arising from altered nociception in the absence of tissue damage or of disease of the somatosensory system.(Kosek et al. 2016, 2021) It is for precisely this reason the most exposed to the recognition deficit, since neither histology nor a conclusive test stands behind the patient’s word. The protracted disputes over the nosological status of fibromyalgia and allied conditions are intelligible in this light: they have been at once epistemic struggles, for the resources to render the experience intelligible, and recognitive struggles, for the standing that only medical legitimacy confers.

It does not follow from any of this that recognition ought to be conditioned upon diagnosis. The revised IASP definition embodies the opposite norm: its accompanying notes hold that a person’s report of pain should be respected as such, irrespective of whether it can be tied to a recognised pathology.(Raja et al. 2020) The norm is sound but, on its own, inert. So long as institutional recognition remains bound to classificatory availability, an injunction to believe the patient corrects the individual credibility judgement while leaving untouched the arrangement that withholds standing from the unclassified. The point holds in the other direction as well. A clinician may withhold, delay, or decline a particular diagnosis while fully recognising the patient and continuing to offer care, follow-up, and treatment. There can be good epistemic, pragmatic, or hermeneutical reasons to resist a premature or reifying label, not least the looping effects already noted, and such restraint is no failure of recognition. That recognition can coexist with the refusal of a label shows that its dependence on diagnosis is a contingent feature of present institutional arrangements, not a conceptual necessity. It is the arrangement, not the individual diagnostic act, that the present analysis identifies as the locus of harm. This is what the heuristic perspective discloses: an exclusively epistemic remedy, addressed to the credibility of testimony, cannot reach a deficit lodged in the institutional binding of recognition to classification, for in that arrangement the epistemic and the recognitive are not two separately soluble problems but one. The inseparability here is of a different order from that of the single testimonial act in Sect. ''Testimony and the Indivisibility of Credibility and Care'' not a constitutive unity but a contingent fusion produced by institutional design, and reformable precisely because contingent (Sect. '' For Clinical Practice'').

### The clinical relationship as intersubjective unity

A third dimension of the encounter lies below the threshold of speech. Drawing on Honneth’s Winnicottian account of primary recognition, Lobb identifies a form of epistemic harm she calls prediscursive epistemic injury, one that operates at the level of embodied, affective attunement rather than explicit testimony. (Lobb 2018) There are, on this view, modes of knowing and being known that precede articulation, among them the patient’s somatic sense of whether they are being attended to with concern or merely processed through a protocol.

In the pain encounter this register carries unusual weight. Attentiveness, or its absence, is conveyed through posture, eye contact, the quality of the silence during history-taking, the pace of questioning. Such micro-behaviours are not mere accompaniments to the exchange of testimony and assessment; they are the medium through which credibility is conferred or withheld and, in the same gesture, the medium through which recognition is extended or denied. The patient who senses that the clinician has already decided meets at once a pre-emption of testimonial assessment and a withdrawal of the attentive concern in which recognition in the sphere of primary relations consists, the two arriving as one.

Research on sex bias in pain management points the same way. When men and women report equivalent pain, the pain attributed to women is liable to be judged lower, the discrepancy ascribed to emotional expressiveness or exaggeration.(Guzikevits et al. 2024) The demographic disparity is one of sex, but the stereotype that mediates it is gendered: the figure of hysteria, with its long medical lineage, is at once an identity prejudice that deflates credibility and a cultural narrative that denies esteem to a particular mode of embodied suffering. Here too the epistemic and the recognitive are not addressed apart, the same stereotype deflating credibility and denying esteem in one stroke.

## Pain as a particularly illuminating case

The argument has been developed throughout with reference to pain, but pain is not the only experience that exhibits the structure described. It belongs to a broader class of what may be called testimonially dependent forms of suffering, experiences whose disclosure to others runs principally or exclusively through the sufferer’s own report. The question is therefore not whether pain is uniquely paradigmatic, but what its place is within this class, and how far the co-constitution thesis travels beyond it.

Three structural features characterise the class. The first is radical privacy: the experience cannot be directly observed, measured, or verified by a third party, so that the epistemic relationship between sufferer and clinician is of necessity a testimonial one. The second is resistance to articulation; Scarry’s claim that pain is world-destroying, that it strains and may overwhelm the expressive resources through which experience is ordinarily shared, names a difficulty that besets the whole class, even if it has been qualified by later scholarship.(Scarry 1987) The third is existential dependence: the sufferer relies on another’s willingness to receive the testimony and respond with care, and retains little capacity for autonomous redress. None of these features is peculiar to pain. They are equally characteristic of psychiatric symptoms, where the same testimonial fragility has been documented (Crichton et al. 2017; Kidd et al. 2025), and of experiences such as trauma and dissociation, which are private, hard to articulate, and dependent on uptake from others.

If the three features are shared, what distinguishes pain is the constellation in which they appear, for in pain they occur largely free of three complications that attend other members of the class. First, the reality of the experience is not in question. With a psychotic experience, the prior question of whether the experience tracks reality is itself live, so that a withholding of credibility may, in some instances, reflect a genuine epistemic difficulty and not only prejudice. This is not to say that such testimony is rightly discounted; the literature on epistemic injustice in psychiatry shows that it frequently is not.(Crichton et al. 2017) The point is analytic: where a veridicality question is independently in play, the co-constitutive structure is harder to isolate, because a deflation of credibility cannot be referred cleanly to prejudice. The report of pain admits no such doubt at the level of experience, since the pain is real as experience whatever its correlate in tissue. On the understanding reflected in the revised IASP definition, a person’s report of their pain is to be respected as such. (Raja et al. 2020) The contrast rests on a substantive though contestable premise, owned here as such: that the experience of pain is self-intimating in a way the veridicality of a perception is not. The doubt described in Sect. ''Diagnostic Language as Epistemic and Recognitive Medium'' attaches not to the existence of the experience but to its medical objectification. It is precisely the misdirection of that doubt, from the reality of the suffering to the absence of a surrogate object, that the present analysis identifies as the prejudicial move.

Second, the testifier’s cognitive standing is, in the central case, less directly in question than where a condition carries the attribution of disordered cognition, an attribution that is itself frequently turned into a ground for discounting testimony, although it functions there as a further site of injustice and not as a warrant; psychiatric conditions, too, rarely admit of a biomarker, and the presumption of unreliability is often built into their very diagnostic criteria. The qualification is needed, since pain that resists objectification is frequently met with a charge of psychogenic origin that approaches such an attribution; but that charge is itself a mode of the prejudicial deflation under analysis, not a separate epistemic warrant for it. The gendered stereotype of emotional exaggeration discussed in Sect. '' The Clinical Relationship as Intersubjective Unity'' is no counter-instance for the same reason: it deflates credibility through prejudice rather than ascribing a competence-defeating disorder of cognition. Third, pain is ordinarily localisable in time and circumstance, where other forms of suffering may be diffuse or pervasive. Because these confounds are absent, the credibility deficit in the pain encounter, where it occurs, cannot be rationalised by appeal to a prior doubt about the reality of the experience or to an imputed disorder of cognition. One source of doubt does persist, the suspicion that a report is insincere and offered for secondary gain. But it is not a confound of the same order, for it impugns the speaker’s honesty rather than the reality or the cognitive credentials of the experience, and it is taken up in Sect. ''For Clinical Practice''. Setting it aside, the deficit stands revealed for what it is, a deflation of credibility bound up with a refusal of recognition. Pain therefore isolates the co-constitutive structure with a clarity, if not an absolute purity, that the more heavily confounded cases do not afford.

Two conclusions follow. Pain is not paradigmatic in the strong sense of being ontologically unique or differing in kind from the other members of its class. The structure it displays is shared; pain is paradigmatic only in the methodological sense of being the case in which that structure is seen most clearly, least obscured by the doubts that cloud the others. To call pain a particularly illuminating case is to claim this methodological privilege and no more. As for generalisation, whether and how far the co-constitution thesis extends to psychiatric illness, trauma, and other invisible suffering is an empirical and conceptual question, not one to be settled in advance. The work on epistemic injustice in psychiatry suggests both genuine parallels and instructive differences.(Crichton et al. 2017; Kidd et al. 2025) There is, moreover, good reason to expect the co-constitutive structure to recur, in modified form, across much of this class. The thesis is accordingly best understood as a research-guiding heuristic, a lens that brings a structure into focus and invites its testing across domains, rather than as a universal theory of the relation between epistemic injustice and recognition.

## Implications

### For social philosophy

For social philosophy, the customary division of labour, with epistemic injustice assigned to social epistemology and the failure of recognition to moral and political philosophy, proves harder to sustain than it appears, at least across testimonially dependent encounters. The perspective offered here is heuristic rather than synthetic: it asserts neither the identity of the two frameworks nor their mutual reducibility, only that separating the epistemic from the recognitive in the clinical encounter exacts descriptive and practical losses. It thereby recasts Fricker’s suggestion that recognition might admit an epistemically specific form (Fricker 2018): the epistemic appears not as a further species to be set beside Honneth’s three, but as a dimension of the recognitive itself, inseparable from it in the encounters at issue. Honneth’s priority of the moral over the epistemic (Honneth 2022) is, on this reading, no rival to it but a theoretically directed version of it. The mapping must be drawn with care, since Honneth’s *moral recognition* answers to the single sphere of rights, whereas the *recognitive* dimension at issue here cuts across all three. The claim is not that the two vocabularies coincide term for term, but that Honneth’s directed implication and the inseparability defended here are two expressions, at different levels, of one refusal to treat the epistemic and the recognitive as independent.

Two questions remain open: the empirical operationalisation of the thesis, given the still-unsettled state of the field (Nielsen et al. 2025), and its generalisability beyond pain. (Crichton et al. 2017; Kidd et al. 2025) Both bear directly on the practical implications that follow.

### For medical ethics

The clearest practical consequence of the co-constitution thesis is for medical ethics, and it can be put plainly: the problem of pain management cannot be addressed within a framework that treats epistemic justice and recognition as separate goods to be pursued independently. Guidelines that enjoin clinicians to believe the patient address the epistemic dimension but may leave the recognitive untouched, since a clinician can accept a pain report as credible and still fail to acknowledge the patient as a suffering subject whose experience commands a response. Interventions built around empathy and compassion address the recognitive dimension but may leave intact the structural epistemic biases, the false beliefs about biological difference and the gendered stereotype of hysteria documented earlier, that systematically deflate the credibility of particular patients. Each strategy, pursued alone, addresses half of a single problem.

What follows is an integrated demand: that credibility assessment and the extension of recognition be understood as aspects of one task rather than two. A clinician’s epistemic practices, the way they listen, the questions they ask, the weight they give the patient’s report against instrumental findings, are already recognitive practices. Failures of recognition are, correspondingly, not merely ethical lapses but epistemic ones, since the clinician who does not acknowledge a patient’s suffering is also less likely to assess their condition accurately. The separation this calls into question is, in the first place, conceptual. It is not claimed that existing training everywhere keeps the two apart, for good communication and ethics teaching often already weaves testimonial sensitivity together with empathy, bias-awareness, and patient-centred attention. The claim is rather that theorising epistemic virtue and recognitive virtue as distinct educational aims misdescribes their relation, and that, where curricula or guidelines do treat them as separable, the heuristic advanced here predicts a characteristic shortfall.

That prediction is, in principle, testable. It does not follow from the heuristic thesis alone, which by itself entails nothing about clinical outcomes; it follows only once that thesis is joined to a further and frankly empirical commitment, that an intervention addressing the unified relation outperforms one addressing a fragment of it. The co-constitution thesis is thus not itself an empirical hypothesis; so conjoined, however, it issues in one, and offers the field a determinate claim to test where its critics have found chiefly conceptual assertion.(Nielsen et al. 2025) The prediction is then clear: interventions targeting only one dimension, credibility-enhancement without recognitive attention or empathy training without correction of the biases that deflate credibility, should prove less effective at improving the care of patients in pain than interventions that address both together.

### For clinical practice

At the level of clinical practice, the diagnostic moment within the clinical encounter is where the epistemic and the recognitive converge most acutely, as Sect. ''Diagnostic Language as Epistemic and Recognitive Medium'' has shown. The act of diagnosis is at once an epistemic achievement and the conferral of standing on a person as one whose suffering is real and deserving of institutional response. Because classification loops back upon the experience it names and upon the patient’s place in the system (Gauld et al. 2025), the design of diagnostic categories and assessment instruments is never nosologically neutral; their structure settles not only what can be known about pain but who can be recognised as a patient in pain. This yields a concrete reform task. If the harm identified here lies in the binding of recognition to classificatory availability, then classification systems, care pathways, and entitlement criteria should be designed so that recognition is not made hostage to the presence of a settled label. The standing, the access to care, and the legitimate occupancy of the sick role that accompany acknowledged illness should extend to well-attested suffering that does not yet, or may never, map onto a discrete diagnostic category.

One objection presses against any such recommendation, and it deserves acknowledgement rather than evasion: the worry that some who report pain do so falsely, for secondary gain. The possibility is real and cannot be wished away. It does not, however, tell against the position defended here. The argument does not enjoin belief in defiance of evidence; it identifies as unjust the withholding of credibility and recognition on the basis of prejudice, or of the mere absence of a lesion. The reflexive appeal to the malingerer is, moreover, not evenly distributed: the suspicion falls more readily on patients already marginalised by gender, race, or the want of an objectifying test, so that the question of who might be lying often performs the very discounting the analysis sets out to expose. To take testimony seriously is not to abandon judgement; it is to refuse a default of suspicion set by prejudice rather than by evidence.

## Conclusion

This article has argued that the relationship between epistemic injustice and the failure of recognition, in the context of pain, is not exhausted by sequential causation but is, in the heuristic sense defended here, one of co-constitution. In the clinical encounter with a person in pain, the epistemic and the recognitive cannot be separated without descriptive and practical loss: to withhold credibility from a sufferer’s testimony is at the same time to deny recognition to that person as a subject whose suffering commands acknowledgement, and to deny recognition is at the same time to commit an epistemic wrong. Pain is a particularly illuminating case of this structure, not because it is unique, but because its privacy, its resistance to articulation, and the sufferer’s dependence on the other’s response converge in it with a clarity that related forms of suffering, attended by further complications, do not afford.

The argument is limited in scope. It has drawn on a selective body of evidence, and further qualitative and quantitative work would be needed to assess the thesis against a wider range of encounters and populations. The practical proposals sketched above, an integrated conception of epistemic and recognitive remedies and the design of classification systems that do not make recognition hostage to diagnosis, are programmatic, and their development is a task for interdisciplinary collaboration between philosophers, clinicians, and patients.

What the argument does accomplish is a shift in how the philosophical stakes of pain are conceived. Pain is not merely an occasion for applying pre-existing frameworks; taken seriously, it tests them. The inseparability of the epistemic and the recognitive in the pain encounter suggests that the conventional division of philosophical labour, epistemology in one place, ethics and social philosophy in another, is less stable than it appears. In the encounter with a person in pain, knowing and acknowledging, believing and caring, are not separate tasks to be discharged independently but aspects of a single, demanding form of human responsiveness.

## Data Availability

Not applicable. No datasets were generated or analysed in the preparation of this article.
